# Beliefs and Behaviors about Breast Cancer Recurrence Risk Reduction among African American Breast Cancer Survivors

**DOI:** 10.3390/ijerph13010046

**Published:** 2015-12-23

**Authors:** Benjamin Ansa, Wonsuk Yoo, Mary Whitehead, Steven Coughlin, Selina Smith

**Affiliations:** 1 Institute of Public & Preventive Health, Georgia Regents University, CJ-2300 1120 15th Street Augusta, GA 30912, USA; wyoo@gru.edu (W.Y.); sesmith@gru.edu (S.S.); 2 College of Dental Medicine, Georgia Regents University, Augusta, GA 30912, USA; 3 SISTAAH Talk Breast Cancer Support Group, Miami, FL 33169, USA; rendo@bellsouth.net; 4 Department of Community Health and Sustainability, Division of Public Health, University of Massachusetts, Lowell, MA 01854, USA; stevecatlanta@aol.com; 5 Department of Family Medicine, Medical College of Georgia, Augusta, GA 30912, USA

**Keywords:** African Americans, breast cancer, recurrence, risk factors, diet, physical activity, body weight, survivorship

## Abstract

A growing body of evidence suggests that breast cancer recurrence risk is linked to lifestyle behaviors. This study examined correlations between breast cancer recurrence, risk reduction beliefs, and related behaviors among African American breast cancer survivors (AA BCSs). Study participants included 191 AA BCSs, mean age = 56.3 years, who completed a lifestyle assessment tool. Most respondents believed that being overweight (52.7%), lack of physical activity (48.7%), and a high fat diet (63.2%) are associated with breast cancer recurrence. Over 65% considered themselves overweight; one third (33.5%) agreed that losing weight could prevent recurrence, 33.0% disagreed, while the remaining 33.5% did not know; and nearly half (47.9%) believed that recurrence could be prevented by increasing physical activity. Almost 90% survivors with BMI < 25 Kg/M^2^ reported no recurrence compared to 75.7% with BMI ≥ 25 Kg/M^2^ (*p* = 0.06); nearly all of the women (99.2%) answered “yes” to seeking professional help to lose weight, 79.7% of which were recurrence-free (*p* = 0.05). These results provide information about AA BCSs’ beliefs and behaviors protective against breast cancer recurrence. Additional research is warranted to determine the effectiveness of educational interventions for AA BCSs that promote consumption of a healthy diet and engaging in regular physical activity.

## 1. Introduction

With advances in screening and treatment, the number of women surviving breast cancer (BC) has continued to grow [1,2]. In the US, there are an estimated 2.8 million breast cancer survivors, accounting for 41% of all female cancer survivors [3]. Despite this increase in survival, women diagnosed with BC, and treated with surgery plus adjuvant therapy have a recurrence risk of 5%–13% [4,5]. Tumor size and axillary lymph node metastasis are the most relevant risk factors for tumor relapse [6,7]. The primary means for reducing recurrence is medical intervention (e.g., chemotherapy, radiation, and endocrine treatment) [8,9]. However, a growing body of evidence suggests that risk of BC recurrence is linked to lifestyle behaviors, (e.g., excessive body weight, weight gain, physical inactivity, inadequate dietary intake, and excessive alcohol use) [10,11,12,13,14,15]. The American Cancer Society [16] and the American Institute for Cancer Research [17] recommend that women with a history of BC eat fruits and vegetables regularly, be physically active, and maintain a healthy body weight.

Maintaining a healthy weight [18,19], performing physical activity [19,20,21], reducing dietary fat consumption [13], consuming fruits and vegetables [19], and limiting alcoholic intake [14] are important lifestyle behaviors that may reduce BC recurrence. Although BC survivors may be aware of the relationship between lifestyle and risk of BC recurrence [22,23], many report poor adherence to cancer prevention strategies aimed at reducing recurrence [24]. Weight gain is a common problem among BC survivors [25,26]. Only about one-third engage in the recommended level of physical activity [18,27], and <18% consume the recommended amounts of fruits and vegetables [1,28]. Failure to adhere to cancer prevention guidelines may be linked to a lack of knowledge of the relationship between lifestyle behaviors and BC recurrence [29].

Compared to white women, African American survivors face disparities in morbidity and mortality [30] and are more likely to be obese, physically inactive, and not consume the recommended daily fruit and vegetable servings [31]. New cases of BC expected to occur in 2013 among African American women were estimated at 27,060 [32], and the time period 2000–2009 witnessed incidence rates increasing slightly among African American women (0.7% per year) and decreasing among white women (1.0% per year) [33]. The overall five-year relative survival rate for BC diagnosed in 2002–2008 was lower among African American women, compared to white women (78% *vs.* 90%) [34]. This difference can be attributed to both later stage at detection and poorer stage-specific survival among African American women; only about 51% of BC diagnosed among African American women are at a local stage, compared to 61% among white women [34].

Few studies have examined beliefs regarding reduction in risk of recurrence and behaviors among BC survivors [29,35,36,37]. The aim of the current study was to examine correlations between BC recurrence and risk reduction beliefs and related behaviors among African American Breast Cancer Survivors (AA BCSs).

## 2. Methods

The research protocol, *Assessing lifestyle modification needs and experiences of AA BCSs*, has been described elsewhere [38]. Briefly, the two-year needs assessment (2013–2015) included a literature review to determine lifestyle modification strategies; a secondary analysis of the 2009–2010 National Health Interview Survey (NHIS) Cancer Control Supplement to examine health-related quality of life (HR-QoL); a lifestyle assessment tool relating weight and BC history, physical activity, and dietary intake; and focus group discussions to determine salient features for inclusion in a lifestyle intervention.

### 2.1. Participants

Participants were members of a BC support group, Survivors Involving Supporters to Take Action in Advancing Health (SISTAAH) Talk, in Miami, Florida. Founded in 1995, SISTAAH Talk was initiated at the University of Miami Sylvester Comprehensive Cancer Center as the area’s first support group for AA BCSs.

### 2.2. Procedure

The Institutional Review Board of Morehouse School of Medicine approved the study. Between January 2013 and December 2014, researchers recruited 300 eligible BC survivors through mailed letters (outlining specific aims of the study) or face-to-face encounters (during monthly support group meetings). To enhance recruitment, the SISTAAH Talk facilitator contacted potential participants to encourage involvement. Following consent, participants (*n* = 240) who were English-speaking/reading and at least one-year post-treatment, completed the assessment tool through various modes (e.g., self-administered on-line or mailed version or facilitator-administered in-person or by telephone interview).

The response rate was 80% of the total support group membership, diagnosed between one and 25 years.

### 2.3. Measures

In addition to capturing socio-demographic variables and BC history, the assessment tool also included scales related to dietary intake, physical activity, weight history, and knowledge about beliefs regarding cancer risks. The present report focuses on socio-demographic factors, BC history, beliefs, and behaviors.

*Socio-demographic variables and breast cancer recurrence*. Demographics assessed included: age (18–34; 35–54; and 55 years of age and older); education (high school or less, college, or graduate); income ($0–$24,000, $25,000–$49,999, or ≥$50,000); marital status (single, married, widowed/divorced); and insurance coverage (none, Medicare, Medicaid, health insurance/HMO). BC diagnosis and treatment history was captured as year of diagnosis, stage at diagnosis, hormone receptor status, type of treatment received, and history of recurrence.

*Breast cancer recurrence risk reduction beliefs.* Participants’ beliefs related to lifestyle (weight, physical activity, and dietary intake) were assessed by determining the extent to which survivors believed that they were overweight, if being overweight influences recurrence risk, and if losing weight prevented a recurrence. For beliefs related to physical activity, participants responded to two questions: (1) “*is a lack of exercise associated with recurrence?*” and (2) “*does increasing physical activity prevent a recurrence?*” To determine potential associations between dietary intake and BC recurrence, participants responded to a question related to a high fat diet and recurrence.

*Behaviors related to reduction in risk of breast cancer recurrence.* Participant behaviors, specifically, physical activity, dietary intake and weight history, were captured through validated scales. The Behavioral Risk Factor Surveillance System (BRFSS) is a cross-sectional health-related telephone survey that collects data on risk behaviors throughout the US [39]. The 2011 BRFSS includes a standard set of questions on current health behaviors. Physical activity is captured for the past 30 days through eight questions about exercise, recreation and physical activity other than regular job duties. Participants were asked 25 questions capturing the different types of foods and beverages consumed in the past 30 days, including meals and snacks consumed at home, at work or school, in restaurants and anyplace else. Weight history was determined based on responses to the National Health and Nutrition Examination Survey (NHANES) [40], a national questionnaire assessing the health and nutritional status of adults and children in the US. The 2009–2010 NHANES weight history scale includes 20-items related to body weight, including self-perception of weight, self-reported weight over the participant’s lifetime, attempted weight loss during the past 12 months, and methods used to try to lose weight and to keep from gaining weight. BC risk reduction behaviors examined included diet-related cancer risk variables (red meat, processed meat, fruits and vegetables, whole grains, sweets, sugar, candy, and junk foods); physical activity (exercise, including strength training); and weight (calculated BMI, attempts at weight loss and prevention of weight gain).

### 2.4. Statistical Analyses

Descriptive statistics were generated to characterize socio-demographic variables, beliefs, and behaviors of BC survivors related to BC recurrence risk, using frequencies and proportions for all categorical data, and to identify relationships between risk reduction beliefs and behaviors and BC recurrence among AA BCSs. Multivariate logistic regression analyses were conducted to simultaneously assess the association of risk reduction beliefs and behavior performance with BC recurrence. Models were conducted for risk reduction beliefs and behavior performance of weight, physical activity and dietary intake, separately. All models were adjusted for age, income, education, marital status, and stage at diagnosis. Odds ratios and related 95% confidence intervals were derived from multivariate analyses. The significance level was set at 0.05, and all tests were two-sided. All statistical analyses were accomplished with SAS version 9.2 (SAS Institute, Cary, NC, USA).

## 3. Results

### 3.1. Study Sample

Of the 240 BC survivors who completed the lifestyle assessment tool, 49 were excluded for failure to complete the weight and height questions; the overall response rate was 80%. A total of 191 AA BCSs with mean age of 56.3 years (standard deviation = 11.4 years and range = 18–55+ years) completed the assessment tool (Table 1). Although 49 of the 240 survivors did not complete BMI information, we did not find any statistically significant differences with respondents who did not have missing information on BMI. Most were ≥55 years old (59.9%) and college educated (62.8%), 39.6% were widowed/divorced, and 36.4% earned an annual income between $25,000 and $49,999. Over 68% presented with stage I or II BC at diagnosis, 88.3% had surgical treatment, 66.0% had radiotherapy and 58.5% had chemotherapy. More than 62% reported BMI values ≥25 Kg/M^2^. There were 149 (79.7%) recurrence-free BCSs and 38 (20.3%) reporting recurrence. The stage at diagnosis was significantly linked to BC recurrence (*p* < 0.0001) followed by BMI (*p* = 0.08).

**Table 1:** Characteristics of African American breast cancer survivors by breast cancer recurrence status.

Variable	Total N (%)	Recurrence N (%)	No Recurrence N (%)	*p*-Value *
**Age (Years)**				0.72
18–34	6 (3.2)	2 (5.3)	4 (2.7)	
35–54	69 (36.9)	14 (36.8)	55 (36.9)	
≥55	112 (59.9)	22 (57.9)	90 (60.4)	
**Income (Annual)**				0.80
$0–$24,999	59 (31.5)	14 (35.9)	45 (30.4)	
$25,000–$49,999	68 (36.4)	13 (33.3)	55 (37.2)	
≥$50,000	60 (32.1)	12 (30.8)	48 (32.4)	
**Education**				0.49
High school or Less	40 (21.3)	11(28.2)	29 (19.5)	
College	118 (62.8)	22 (56.4)	96 (64.4)	
Graduate	30 (15.9)	6 (15.4)	24 (16.1)	
**Marital Status**				0.93
Single	43 (23.0)	9 (23.7)	34 (22.8)	
Married	70 (37.4)	15 (39.5)	55 (36.9)	
Widowed/Divorced	74 (39.6)	14 (36.8)	60 (40.3)	
**Stage at Diagnosis**				**<0.0001**
Stage I	76 (41.1)	11 (28.2)	65 (44.5)	
Stage II	50 (27.0)	7 (17.9)	43 (29.5)	
Stage IIIA, IIIB &IV	40 (21.6)	19 (48.7)	21 (14.4)	
Don’t Know	19 (10.3)	2 (5.1)	17 (11.6)	
**BC Treatment**				
Chemotherapy	110 (58.5)	27 (69.2)	83 (55.7)	0.13
No Chemotherapy	78 (41.5)	12 (30.8)	66 (44.3)	
Surgery	166 (88.3)	37 (94.9)	129 (86.6)	0.15
No Surgery	22 (11.7)	2 (5.1)	20 (13.4)	
Radiation	124 (66.0)	27 (69.2)	97 (65.1)	0.63
No Radiation	64 (34.0)	12 (30.8)	52 (34.9)	
Hormone	58 (30.8)	11 (28.2)	47 (31.5)	0.69
No Hormone	130 (69.2)	28 (71.8)	102 (68.5)	
**BMI**				0.08
Healthy (BMI = 18.5–24.9 Kg/M^2^)	71 (37.8)	9 (23.1)	62 (41.6)	
Overweight (BMI = 25–29.9 Kg/M^2^)	60 (31.9)	17 (43.6)	43 (28.9)	
Obese (BMI ≥ 30 Kg/M^2^)	57 (30.3)	13 (33.3)	44 (29.5)	

Some variables reflect an *n* < 191 due to missing data for those variables. * *p*-value < 0.05 is significant.

### 3.2. Beliefs Regarding Risk Reduction for BC Recurrence

For beliefs regarding risk reduction for BC recurrence, participants responded to questions concerning weight, physical activity, and dietary intake (Table 2). Over 65% of survivors considered themselves overweight, and many believed that being overweight (52.7%), lack of physical activity (48.7%), and a high-fat diet (63.2%) are associated with BC recurrence. Almost half of the women (47.9%) believed that recurrence could be prevented by increasing physical activity. One third (33.5%) agreed that losing weight could prevent BC recurrence; 33.0% disagreed; and 33.5% did not know. The belief about the association between being overweight and BC recurrence correlated significantly with the recurrence status of the survivors (*p* = 0.04). More than 80% of survivors who believed that being overweight was associated with BC recurrence were recurrence-free, compared to 68.4% who did not believe this.

**Table 2:** Breast cancer recurrence risk reduction beliefs by breast cancer recurrence status.

Lifestyle Variable	Total N (%)	BC Recurrence		*p*-Value *
		Yes	No (Free)	
**Weight**				
Do you consider yourself overweight now?				0.29
Overweight and obese (BMI ≥ 25 Kg/M^2^)	124 (66.3)	30 (76.9)	94 (63.5)	
Underweight (BMI < 18.5 Kg/M^2^)	6 (3.2)	0 (0.0)	6 (4.1)	
Healthy weight ( BMI = 18.5–24.9 Kg/M^2^)	54 (28.9)	9 (23.1)	45 (30.4)	
Don’t Know	3 (1.6)	0 (0.0)	3 (2.0)	
Is being overweight associated with BC recurrence?				**0.04**
Yes	98 (52.7)	18 (46.2)	80 (54.4)	
No	57 (30.6)	18 (46.2)	39 (26.5)	
Don’t Know	31 (16.7)	3 (7.6)	28 (19.1)	
Will BC recurrence be prevented by losing weight?				0.44
Yes	63 (33.5)	13 (33.3)	50 (33.6)	
No	62 (33.0)	10 (25.7)	52 (34.9)	
Don’t know	63 (33.5)	16 (41.0)	47 (31.5)	
**Physical Activity**				
Is lack of physical activity/exercise associated with BC recurrence?				0.18
Yes	91 (48.7)	16 (42.1)	75 (50.3)	
No	43 (23.0)	13 (34.2)	30 (20.1)	
Don’t Know	53 (28.3)	9 (23.7)	44 (29.6)	
Does increasing physical activity/exercise prevent BC recurrence?				0.18
Yes	89 (47.9)	14 (35.9)	75 (51.0)	
No	41 (22.0)	9 (23.1)	32 (21.8)	
Don’t Know	56 (30.1)	16 (41.0)	40 (27.2)	
**Dietary Intake**				**0.04**
Is high fat diet associated with BC recurrence?				
Yes	117 (63.2)	24 (61.5)	93 (63.7)	
No	27 (14.6)	10 (25.7)	17 (11.6)	
Don’t Know	41 (22.2)	5 (12.8)	36 (24.7)	

Some variables reflect an *n* < 191 due to missing data for those variables. * *p*-value < 0.05 is significant.

The correlation was significant between the belief that a high fat diet is associated with BC recurrence and recurrence status (*p* = 0.04); 79.5% of women who answered “yes” were recurrence-free, compared to 63.0% who answered “no”. Although differences were not statistically significant in either case, survivors who also remained recurrence-free included those with normal body weight (83.3% *vs.* 75.8% overweight (*p* = 0.29)); those who responded “yes” to the question that lack of exercise is associated with BC recurrence (82.4% *vs.* 69.8% responders who said “no” (*p* = 0.18)); and those who responded “yes” to the question that increasing physical activity will prevent BC recurrence (84.3% *vs.* 78.0% responders who said “no” (*p* = 0.18)).

### 3.3. Behaviors Related to Reduction in Risk of BC Recurrence

BMI values and seeking professional help to lose weight were associated (not significantly) with BC recurrence (Table 3). Almost 90% of survivors with BMI values < 25 Kg/M^2^ reported no recurrence (compared to 75.7% with BMI values ≥ 25 Kg/M^2^ (*p* = 0.06)). Nearly all of the women (99.2%) answered “yes” to seeking professional help to lose weight, 79.7% of these were recurrence-free (*p* = 0.05); survivors who reported ‘no’ to eating red meat and processed meat were all recurrence free (*p* = 0.11; *p* = 0.20 respectively). Other behaviors in the three lifestyle categories were also not significantly correlated to BC recurrence, and all of the survivors surveyed reported ‘yes’ to eating fruits or vegetables, salad, and whole grains.

**Table 3:** Breast cancer recurrence risk reduction behaviors by breast cancer recurrence status.

Lifestyle Variable	Total N (%)	BC Recurrence		*p*-Value *
		Yes	No (Free)	
**Weight**				
BMI (Kg/M^2^)				0.06
BMI <25 Kg/M^2^	71 (40.8)	9 (26.5)	62 (44.3)	
BMI ≥25 Kg/M^2^	103 (59.2)	25 (73.5)	78 (55.7)	
Have you ever tried to lose weight?				0.14
Yes	155 (82.4)	29 (74.4)	126 (84.6)	
No	33 (17.6)	10 (25.6)	23 (15.4)	
Did you seek help from a professional to lose weight?				0.05
Yes	133 (99.2)	27 (96.4)	106 (100.0)	
No	1 (0.8)	1 (3.6)	0 (0.0)	
**Physical Activity**				
Did you participate in any physical activities/exercises in the past month?				0.47
Yes	127 (69.8)	24 (64.9)	103 (71.0)	
No	55 (30.2)	13 (35.1	42 (29.0)	
Did you do muscle strengthening in the past month?				0.82
Yes	117 (68.0)	23 (69.7)	94 (67.6)	
No	55 (32.0)	10 (30.3)	45 (32.4)	
**Dietary Intake**				
Did you eat red meat in the past month?				0.11
Yes	169 (94.9)	37 (100.0)	132 (93.6)	
No	9 (5.1)	0 (0.0)	9 (6.4)	
Did you eat processed meat in the past month?				0.20
Yes	172 (96.6)	37 (100.0)	135 (95.7)	
No	6 (3.4)	0 (0.0)	6 (4.3)	
Did you eat fruits or vegetables in the past month?				(None)
Yes	181 (100.0)	39 (100.0)	142 (100.0)	
Did you eat salad in the past month?				(None)
Yes	184 (100.0)	39 (100.0)	145 (100.0)	
Did you eat whole grain in the past month?				(None)
Yes	178 (100.0)	35 (100.0)	143 (100.0)	
Did you eat sweets, sugar, candy in the past month?				0.39
Yes	173 (98.3)	34 (100.0)	139 (97.9)	
No	3 (1.7)	0 (0.00)	3 (2.1)	

Some variables reflect an *n* < 191 due to missing data for those variables. * *p*-value < 0.05 is significant.

The results from the models of the multivariate logistic regression analyses are compared in Table 4. The odds ratio (OR) for BC recurrence among survivors who answered “yes” (compared to those who answered “no”) to the question “*Is being overweight associated with BC recurrence?*” was 0.90 (95% CI = 0.51, 1.59), and 2.01 (95% CI = 0.84, 4.82) for survivors with BMI values ≥ 25 Kg/M^2^ (compared to those with BMI values < 25 Kg/M^2^). Though neither of the odds ratios is statistically significant, those who acknowledged that being overweight is associated with BC recurrence were themselves less likely to have BC recurrence. Responders who believed that lack of physical activity/exercise is associated with BC recurrence were less likely to have recurrence than those who did not believe (OR = 0.76 (95% CI = 0.56, 1.03)); and those who participated in any physical activities/exercises in the past month were less likely to have a recurrence (OR = 0.88 (95% CI = 0.39, 2.02)). Those who believed that a high-fat diet is associated with BC recurrence were less likely to report a recurrence (OR = 0.82 (95% CI = 0.51, 1.32)). The OR and *p*-value could not be calculated for survivors who responded to the question “*Did you eat red meat (beef, pork, ham or sausage) in the past month?*” because those who did not eat red meat all reported being recurrence-free.

**Table 4:** Relationship between breast cancer recurrence and risk reduction beliefs and behaviors: multiple logistic regression analyses.

Factors	Breast Cancer Recurrence		
	Weight OR (95% CI)	Physical activity OR (95% CI)	Dietary Intake OR (95% CI)
Belief *	0.90 (0.51, 1.59)	0.76 (0.56, 1.03)	0.82 (0.51, 1.32)
Behavior **	2.01 (0.84, 4.82)	0.88 (0.39, 2.02)	-
Age	0.67 (0.33, 1.43)	0.76 (0.38, 1.52)	0.77 (0.40, 1.50)
Income	0.77 (0.41, 1.40)	0.81 (0.46, 1.44)	0.85 (0.49, 1.46)
Education	0.56 (0.25, 1.26)	0.84 (0.39, 1.81)	0.77 (0.37, 1.58)
Marital status	0.69 (0.39, 1.24)	0.86 (0.50, 1.48)	0.98 (0.58, 1.66)
Stage at diagnosis	1.27 (0.84, 1.90)	1.31 (0.90, 1.90)	1.44 (1.00, 2.08)

* Questions relating to beliefs: Weight: Is being overweight associated with BC recurrence? Physical activity: Is lack of physical activity/exercise associated with BC recurrence? Dietary intake: Is a high fat diet associated with BC recurrence? ** Items/Questions relating to behaviors: Weight: Body mass index Physical activity: Did you participate in any physical activities/exercises in the past month? Dietary intake: Did you eat red meat (beef, pork, ham or sausage) in the past month? OR: Odds Ratio. CI: Confidence Interval.

## 4. Discussion 

Concerning the beliefs and behaviors of AA BCS, the current study provides information regarding lifestyle factors that are likely to protect against BC recurrence. Physical activity and eating a healthy diet that includes plenty of fruits and vegetables, whole grains, and little or no red meat (or other sources of saturated fat) have been shown to protect against several chronic diseases [16,17]. Although many of the women in the current study were aware that physical activity and a proper diet are likely to protect against BC recurrence, the findings underscore the need for educational interventions to improve AA BCS knowledge and practices associated with reduced risk of BC and other chronic illnesses. For example, 66.3% of the women considered themselves to be overweight, but over half (52.1%) did not believe that, by increasing physical activity, BC recurrence could be prevented. Only one third (33.5%) believed that weight loss could prevent BC recurrence and another third (33.5%) did not know if this is true. In a similar study by O’Neill S.C. *et al.* [37], where 80% of the responders were white women, and 14% black, about 62% of women reported that they did not have a healthy body weight despite their awareness that maintaining a healthy weight could reduce risk. About one-third of women reported that they did not engage in the recommended behaviors for physical activity (37%) or fruit and vegetable consumption (36%), despite their awareness that these behaviors potentially reduce risk. Additionally, the same study revealed that adherence to healthy behavior recommendations was higher for women who were white, college-educated, and had higher incomes. Burris, J.L. *et al.* [31] revealed that behaviors most commonly endorsed by BC survivors as being effective at reducing BC recurrence risk were those for which empirical evidence supports a linkage (e.g., exercising, maintaining a healthy weight, and diet); and behavior performance was positively correlated with beliefs for most of the itemized behaviors, with the lone exception being “avoid eating meat of any kind”. The study by Weiner, J.G. *et. al.* [36] showed that a small proportion of survivors had a positive correlation between self-reported dietary and exercise behaviors and their perceived benefits of eating fruits and vegetables and exercising; and also that stage at diagnosis was a determinant of BC risk reduction belief and behavior performance. The majority of survivors with early stage diagnosis did not agree that eating at least five servings of fruits and vegetables per day would reduce their risk of breast cancer recurrence.

In the Women’s Healthy Eating and Living (WhEL) Study [41,42], 45% of AA BCS (*n* = 118, median age = 49 years) were obese, which is somewhat higher than the 30% prevalence of obesity observed in the current study. In a Sisters Network, Inc. Study of African American breast cancer survivors (*n* = 470, mean age = 54 years), 47% of the participants were obese [43]. These differences in the prevalence of obesity across studies may reflect differences in physical activity, diet, or environmental influences such as proximity to areas for recreational activity. The present results support the value of including health education on diet, physical activity, and maintaining proper weight in best practices developed for community organizations that provide support for BC survivors [44]. While BC patients may receive health education from their providers or as part of cancer survivorship care plans, the findings of the current study suggest that many AA BCS are not receiving this important information from their oncologists or primary care providers. It is important to note that BC survivors are heterogeneous with respect to their survivorship care needs. Some survivors may be unable to participate in some forms of exercise (e.g., jogging or bicycling) due to physical limitations, chronic pain, or co-morbid conditions.

BC survivors often want to know what they can do to reduce their chances of having a recurrence or worsening of their illness and how to improve their overall physical and psychological health. Eating a healthy diet and regularly exercising can empower BC survivors to gain some control over their health and to improve their quality of life. Lifestyle interventions that promote physical activity and improved diet among overweight and obese cancer survivors provide benefits in physical functioning, body weight, and quality-of-life [45]. Among survivors, a healthy diet is associated with reduced cancer-related fatigue and a reduced risk of non-BC mortality [46].

With respect to limitations, the current study is cross-sectional and based upon self-reported information. Nevertheless, questions on physical activity and diet were in the assessment tool that have previously been used in national surveys conducted for the general US population. Due to differences in culture or language, the generalizability of the findings to other groups of women (e.g., Haitian American or Brazilian-American BCSs) is uncertain.

## 5. Conclusions

Many AA BCSs are overweight or obese, and do not engage in recurrence risk reduction behaviors. Additional research is warranted to examine the effects of life-style interventions among AA BCSs who, in general, have a poorer survival than their white counterparts. For AA BCS, rigorously-designed, longitudinal studies are needed to determine the effectiveness of innovative educational interventions that promote consumption of a healthy diet and regular physical activity [47]. Such health promotion interventions should be culturally-appropriate and tailored to meet the needs of AA survivors. Ideally, researchers should work collaboratively with survivors and family members to ensure that interventions that are developed and tested are relevant and practical [44].

## References

[1] International Agency for Research on Cancer (IARC) Breast Cancer. Estimated Incidence, Mortality and Prevalence Worldwide in 2012.

[2] DeSantis C., Ma J., Bryan L., Jemal A. (2014). Breast cancer statistics, 2013. CA Cancer J. Clin..

[3] American Cancer Society (ACS) Cancer Treatment & Survivorship Facts & Figures 2014–2015. http://www.cancer.org/research/cancerfactsstatistics/survivor-facts-figures.

[4] Bosco J.L., Lash T.L., Prout M.N., Buist D.S., Geiger A.M., Haque R., Wei F., Silliman R.A. (2009). Breast cancer recurrence in older women five to ten years after diagnosis. Cancer Epidemiol. Biomark. Prev..

[5] Brewster A.M., Hortobagyi G.N., Broglio K.R., Kau S.-W., Santa-Maria C.A., Arun B., Buzdar A.U., Booser D.J., Valero V., Bondy M. (2008). Residual risk of breast cancer recurrence 5 years after adjuvant therapy. J. Natl. Cancer Inst..

[6] Ustaalioglu B.O., Balvan O., Bilici A., Develi A., Aliustaoglu M., Vardar F., Erkol B. (2015). The differences of clinicopathological factors for breast cancer in respect to time of recurrence and effect on recurrence-free survival. Clin. Transl. Oncol..

[7] Pogoda K., Niwińska A., Murawska M., Pieńkowski T. (2013). Analysis of pattern, time and risk factors influencing recurrence in triple-negative breast cancer patients. Med. Oncol..

[8] Early Breast Cancer Trialists’ Collaborative Group (EBCTCG) (2005). Effects of chemotherapy and hormonal therapy for early breast cancer on recurrence and 15-year survival: An overview of the randomised trials. Lancet.

[9] Fisher B., Bryant J., Dignam J.J., Wickerham D.L., Mamounas E.P., Fisher E.R., Margolese R.G., Nesbitt L., Paik S., Pisansky T.M. (2002). Tamoxifen, radiation therapy, or both for prevention of ipsilateral breast tumor recurrence after lumpectomy in women with invasive breast cancers of one centimeter or less. J. Clin. Oncol..

[10] Kelly K.M., Bhattacharya R., Dickinson S., Hazard H. (2015). Health Behaviors Among Breast Cancer Patients and Survivors. Cancer Nurs..

[11] Chlebowski R.T., Aiello E., McTiernan A. (2002). Weight loss in breast cancer patient management. J. Clin. Oncol..

[12] Friedenreich C.M., Gregory J., Kopciuk K.A., Mackey J.R., Courneya K.S. (2009). Prospective cohort study of lifetime physical activity and breast cancer survival. Int. J. Cancer.

[13] Chlebowski R.T., Blackburn G.L., Thomson C.A., Nixon D.W., Shapiro A., Hoy M.K., Goodman M.T., Giuliano A.E., Karanja N., McAndrew P. (2006). Dietary fat reduction and breast cancer outcome: Interim efficacy results from the Women’s Intervention Nutrition Study. J. Natl. Cancer Inst..

[14] Kwan M.L., Kushi L.H., Weltzien E., Tam E.K., Castillo A., Sweeney C., Caan B.J. (2010). Alcohol consumption and breast cancer recurrence and survival among women with early-stage breast cancer: The life after cancer epidemiology study. J. Clin. Oncol..

[15] Fedele P., Orlando L., Schiavone P., Quaranta A., Lapolla A.M., de Pasquale M., Ardizzone A., Bria E., Sperduti I., Calvani N. (2014). BMI variation increases recurrence risk in women with early-stage breast cancer. Future Oncol..

[16] Rock C.L., Doyle C., Demark-Wahnefried W., Meyerhardt J., Courneya K.S., Schwartz A.L., Bandera E.V., Hamilton K.K., Grant B., McCullough M. (2012). Nutrition and physical activity guidelines for cancer survivors. CA Cancer J. Clin..

[17] American Institute for Cancer Research (AICR) Recommendations for Cancer Prevention. http://www.aicr.org/reduce-your-cancer-risk/recommendations-for-cancer-prevention/.

[18] Irwin M.L., Fabian C., McTiernan A. (2015). Risk Reduction from Weight Management and Physical Activity Interventions. Improving Outcomes for Breast Cancer Survivors.

[19] Kroenke C.H., Chen W.Y., Rosner B., Holmes M.D. (2005). Weight, weight gain, and survival after breast cancer diagnosis. J. Clin. Oncol..

[20] Holick C.N., Newcomb P.A., Trentham-Dietz A., Titus-Ernstoff L., Bersch A.J., Stampfer M.J., Baron J.A., Egan K.M., Willett W.C. (2008). Physical activity and survival after diagnosis of invasive breast cancer. Cancer Epidemiol. Biomark. Prev..

[21] Holmes M.D., Chen W.Y., Feskanich D., Kroenke C.H., Colditz G.A. (2005). Physical activity and survival after breast cancer diagnosis. JAMA.

[22] Mellon S., Gold R., Janisse J., Cichon M., Tainsky M.A., Simon M.S., Korczak J. (2008). Risk perception and cancer worries in families at increased risk of familial breast/ovarian cancer. Psychooncology.

[23] Partridge A., Adloff K., Blood E., Dees E.C., Kaelin C., Golshan M., Ligibel J., de Moor J.S., Weeks J., Emmons K. (2008). Risk perceptions and psychosocial outcomes of women with ductal carcinoma *in situ*: Longitudinal results from a cohort study. J. Natl. Cancer Inst..

[24] Blanchard C.M., Courneya K.S., Stein K. (2008). Cancer survivors’ adherence to lifestyle behavior recommendations and associations with health-related quality of life: Results from the American Cancer Society’s SCS-II. J. Clin. Oncol..

[25] Vance V., Mourtzakis M., McCargar L., Hanning R. (2011). Weight gain in breast cancer survivors: Prevalence, pattern and health consequences. Obes. Rev..

[26] Gross A.L., May B.J., Axilbund J.E., Armstrong D.K., Roden R.B., Visvanathan K. (2015). Weight Change in Breast Cancer Survivors Compared to Cancer-Free Women: A Prospective Study in Women at Familial Risk of Breast Cancer. Cancer Epidemiol. Biomark. Prev..

[27] Irwin M.L., Crumley D., McTiernan A., Bernstein L., Baumgartner R., Gilliland F.D., Kriska A., Ballard-Barbash R. (2003). Physical activity levels before and after a diagnosis of breast carcinoma. Cancer.

[28] Pierce J.P., Stefanick M.L., Flatt S.W., Natarajan L., Sternfeld B., Madlensky L., Al-Delaimy W.K., Thomson C.A., Kealey S., Hajek R. (2007). Greater survival after breast cancer in physically active women with high vegetable-fruit intake regardless of obesity. J. Clin. Oncol..

[29] Burris J.L., Jacobsen P.B., Loftus L.S., Andrykowski M.A. (2012). Breast cancer recurrence risk reduction beliefs in breast cancer survivors: Prevalence and relation to behavior. Psychooncology.

[30] Centers for Disease Control and Prevention (CDC) Breast Cancer 2012. http://www.cdc.gov/cancer/breast/statistics/state.htm.

[31] American Cancer Society (ACS) Cancer Facts and Figures 2015. http://www.cancer.org/acs/groups/content/@editorial/documents/document/acspc-044552.pdf.

[32] American Cancer Society (ACS) Cancer Facts and Figures for African Americans 2013–2014. http://www.cancer.org/acs/groups/content/@epidemiologysurveilance/documents/document/acspc-036921.pdf.

[33] Ravdin P.M., Cronin K.A., Howlader N., Berg C.D., Chlebowski R.T., Feuer E.J., Edwards B.K., Berry D.A. (2007). The decrease in breast-cancer incidence in 2003 in the United States. N. Engl. J. Med..

[34] Howlader N., Noone A., Krapcho M., Neyman N., Aminou R., Altekruse S., Kosary C., Ruhl J., Tatalovich Z., Cho H. (2012). SEER Cancer Statistics Review, 1975–2009 (Vintage 2009 Populations).

[35] Stolley M.R., Sharp L.K., Wells A.M., Simon N., Schiffer L. (2006). Health behaviors and breast cancer: Experiences of urban African American women. Health Educ. Behav..

[36] Weiner J.G., Jordan T.R., Thompson A.J., Fink B.N. (2010). Analysis of the relationship between diet and exercise beliefs and actual behaviors among breast cancer survivors in Northwest Ohio. Breast Cancer.

[37] O’Neill S.C., DeFrank J.T., Vegella P., Richman A.R., Henry L.R., Carey L.A., Brewer N.T. (2013). Engaging in health behaviors to lower risk for breast cancer recurrence. PLoS ONE.

[38] Smith S.A., Claridy M.D., Whitehead M.S., Sheats J.Q., Yoo W., Alema-Mensah E.A., Ansa B.E., Coughlin S.S. (2015). Lifestyle Modification Experiences of African American Breast Cancer Survivors: A Needs Assessment. JMIR Cancer.

[39] Behavioral Risk Factor Surveillance System (BRFSS). http://www.cdc.gov/brfss/data_documentation/index.htm.

[40] National Health and Nutrition Examination Survey (NHANES). http://www.cdc.gov/nchs/nhanes.htm.

[41] Paxton R.J., Phillips K.L., Jones L.A., Chang S., Taylor W.C., Courneya K.S., Pierce J.P. (2012). Associations among physical activity, body mass index, and health-related quality of life by race/ethnicity in a diverse sample of breast cancer survivors. Cancer.

[42] Paxton R.J., Jones L.A., Chang S., Hernandez M., Hajek R.A., Flatt S.W., Natarajan L., Pierce J.P. (2011). Was race a factor in the outcomes of the women’s health eating and living study?. Cancer.

[43] Paxton R.J., Taylor W.C., Chang S., Courneya K.S., Jones L.A. (2013). Lifestyle behaviors of African American breast cancer survivors: A Sisters Network, Inc. study. PLoS ONE.

[44] Alfano C.M., Smith T., de Moor J.S., Glasgow R.E., Khoury M.J., Hawkins N.A., Stein K.D., Rechis R., Parry C., Leach C.R. (2014). An action plan for translating cancer survivorship research into care. J. Natl. Cancer Inst..

[45] Demark-Wahnefried W., Morey M.C., Sloane R., Snyder D.C., Miller P.E., Hartman T.J., Cohen H.J. (2012). Reach out to enhance wellness home-based diet-exercise intervention promotes reproducible and sustainable long-term improvements in health behaviors, body weight, and physical functioning in older, overweight/obese cancer survivors. J. Clin. Oncol..

[46] Izano M.A., Fung T.T., Chiuve S.S., Hu F.B., Holmes M.D. (2013). Are diet quality scores after breast cancer diagnosis associated with improved breast cancer survival?. Nutr. Cancer.

[47] Coughlin S.S., Yoo W., Whitehead M.S., Smith S.A. (2015). Advancing breast cancer survivorship among African-American women. Breast Cancer Res. Treat..

